# Characteristics, Biodiversity, and Cultivation Strategy of Low Nucleic Acid Content Bacteria

**DOI:** 10.3389/fmicb.2022.900669

**Published:** 2022-06-15

**Authors:** Wei Hu, Hui Zhang, Xiaowen Lin, Ruidan Liu, Mark Bartlam, Yingying Wang

**Affiliations:** ^1^Key Laboratory of Pollution Processes and Environmental Criteria (Ministry of Education), Tianjin Key Laboratory of Environmental Remediation and Pollution Control, College of Environmental Science and Engineering, Nankai International Advanced Research Institute (Shenzhen Futian), Nankai University, Tianjin, China; ^2^State Key Laboratory of Medicinal Chemical Biology, College of Life Sciences, Nankai International Advanced Research Institute (Shenzhen Futian), Nankai University, Tianjin, China

**Keywords:** physical characteristics, terminologies, diversity, functions, cultivation strategy, LNA bacteria

## Abstract

Low nucleic acid content (LNA) bacteria are ubiquitous and estimated to constitute 20%–90% of the total bacterial community in marine and freshwater environment. LNA bacteria with unique physiological characteristics, including small cell size and small genomes, can pass through 0.45-μm filtration. The researchers came up with different terminologies for low nucleic acid content bacteria based on different research backgrounds, such as: filterable bacteria, oligotrophic bacteria, and low-DNA bacteria. LNA bacteria have an extremely high level of genetic diversity and play an important role in material circulation in oligotrophic environment. However, the majority of LNA bacteria in the environment remain uncultivated. Thus, an important challenge now is to isolate more LNA bacteria from oligotrophic environments and gain insights into their unique metabolic mechanisms and ecological functions. Here, we reviewed LNA bacteria in aquatic environments, focusing on their characteristics, community structure and diversity, functions, and cultivation strategies. Exciting future prospects for LNA bacteria are also discussed.

## Introduction

Low nucleic acid content (LNA) bacteria exist widely in the natural environments, including oceans, lakes, rivers, springs, groundwater, drinking water, greenland glacier ice core, wetlands and sediments, ranging from 20% to 90% of the total bacterial community in marine and freshwater environment (Cottrell and Kirchman, 2000; Servais et al., 2003; Longnecker et al., 2005, 2006; Miteva and Brenchley, 2005; Suzina et al., 2011; Liu et al., 2017, 2018; Proctor et al., 2018; Eswaran and Khandeparker, 2021). Microbiologists have given different definitions and names to LNA bacteria based on their characteristics, such as filterable bacteria (Wang et al., 2007), low-DNA bacteria (Gasol et al., 1999). In the field of microbiology, filtration through 0.45-μm pore size filters is a common method to isolate and enrich LNA bacteria from aqueous samples (Wang et al., 2009). Here, we define the bacteria with the characters of fewer than 0.1 μm^3^ in volume, can pass through 0.45-μm microporous membrane, low DNA content showed in flow cytometry (FCM) as LNA bacteria. All bacteria with synonymous names that fall under the definition provided are considered LNA bacteria.

LNA bacteria were initially considered to be inactive, dead, fragment cells (Gasol et al., 1999) and high nucleic acid content (HNA) bacteria were assumed to be the most active group of heterotrophic bacteria in different marine ecosystems (Gasol and Del Giorgio, 2000). With the development of technology, investigators found that LNA bacteria may represent the most active and predominant bacterioplankton community in oligotrophic environments (Zubkov et al., 2001; Jochem et al., 2004; Longnecker et al., 2005). LNA bacteria with a simplified metabolism, efficient substrate uptake system, and special cellular membrane constitution (Liu et al., 2017), can respond quickly to various stressful environment conditions (Ramseier et al., 2011). Thus, it has been proposed that LNA bacteria can be used as bio-indicators to reflect the water quality (Sharuddin et al., 2018; Santos et al., 2019).

LNA bacteria, including species of free-living and parasitic bacteria, have an extremely high level of diversity. Phylogenetic analysis showed that the Alphaproteobacteria SAR11 clade (Mary et al., 2006; Schattenhofer et al., 2011), Gammaproteobacteria SAR86 clade (Zubkov and Sleigh, 1998; Zubkov et al., 2002), Betaproteobacteria, Firmicutes and Fibrobacter (Vila-Costa et al., 2012) were dominant in LNA bacteria populations in marine environments. In freshwater, Actinobacteria Gammaproteobacteria, and Deltaproteobacteria were the dominant phyla in LNA bacterial operational taxonomic units (OTUs; Proctor et al., 2018; Song et al., 2019a). Additionally, it was reported that many LNA bacteria attributed to candidate phyla radiation (CPR) in freshwater ecosystems (Proctor et al., 2018). High-throughput sequencing based on molecular markers and complex metagenomic methods have suggested that LNA bacteria play important roles in the biochemical cycles in marine and freshwater environment (Giovannoni, 2017; Henson et al., 2018; Song et al., 2019a). Whereas the majority of LNA bacteria remain uncultivated under laboratory conditions, the recognition of the physiological growth characteristics and ecological functions of LNA bacteria are still limited.

In this review, we provide an in-depth analysis on LNA bacteria focusing on the following aspects: (1) terminologies of LNA bacteria; (2) the characteristics of LNA bacteria; (3) the community structure and diversity of LNA bacteria; (4) the impact of environmental factors on LNA bacterial community; (5) the functions of LNA bacteria; (6) the cultivation strategy of LNA bacteria.

## Overview of the Terminologies of LNA Bacteria

When considering LNA bacteria, it is important to note the importance of the terms used. Different terminologies and the precise definitions of the terms of small-sized have been widely discussed (Duda et al., 2012; Ghuneim et al., 2018; Nakai, 2020). From the point of view of bacterial species, terminologies can potentially overlap as a single strain can have one or more of these characteristics. Based on the descriptions used in the literature, we summarized the terms for LNA bacteria and their relationships, as shown in Table 1. The terminologies in Table 1 were defined mainly based on three different characteristics of LNA bacteria.

**Table 1:** Description of terms relating to LNA and small-sized bacteria.

Terminology	Characteristics	References
**LNA bacteria**		
Group I cell	Low DNA; Dominate in phytoplankton-poor waters.	Li et al., 1995
Low-DNA bacteria (LDNA)	Low DNA (LDNA) content; Be considered to “dead” bacteria.	Gasol et al., 1999
Low nucleic acid-content bacteria (LNA)	Low nucleic acid content (LNA); Can pass through 0.45-μm filter membrane.	Lebaron et al., 2001; Wang et al., 2009
Filterable bacteria	Can pass through 0.45-μm aperture and smaller aperture filter membrane.	Tabor et al., 1981
**Small-sized bacteria**		
Ultramicrobacteria (UMB)	Less than 0.3 μm in diameter; Cells with consistently small size (<0.1 μm^3^) named obligate UMB; Cells containing a small proportion of larger size (>0.1 μm^3^) named facultative UMB; Small size of the genome (0.58–3.2 Mb).	Duda et al., 2012; Nakai, 2020
Ultramicrocells (UMC)	0.15–0.4 μm in diameter; Nano cells, Dwarf cells, Midget cells, Nanosized cells, Lilliputanian cells.	Duda et al., 2012
Ultra-small bacteria (USB)	Can pass through 0.2-μm filter membrane.	Luef et al., 2015
Nano-sized bacteria	0.05–0.4 μm; Fewer than 0.1 μm^3^ in volume.	Ghuneim et al., 2018

### Nucleic Acid Content

**Figure 1:**
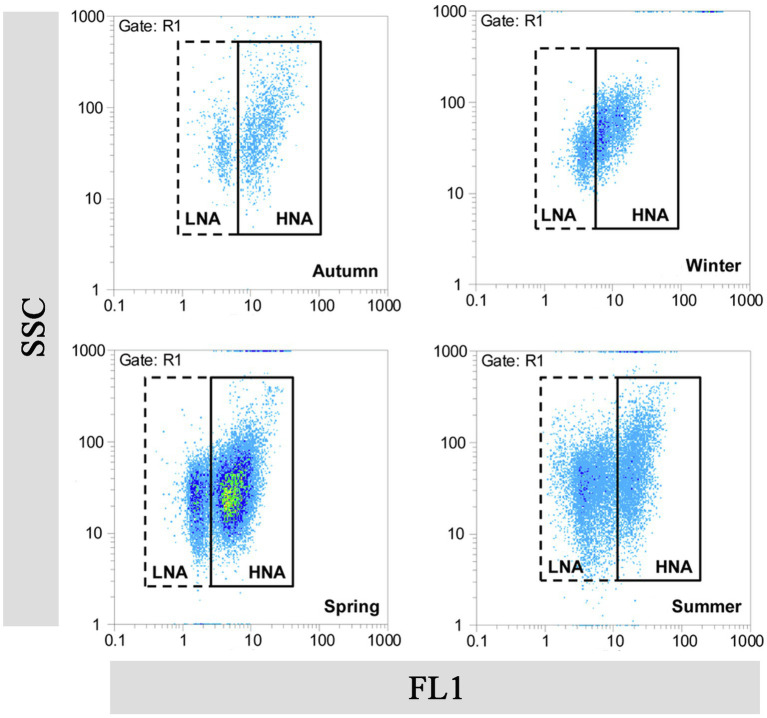
Flow cytogram fingerprint of LNA and HNA bacteria in the Haihe River. This figure is cited from the authors (Liu et al., 2016). FL1, fluorescence intensity; SSC, sideward scatter.

FCM, which is based on fluorescent nucleic acid-staining technologies and independent of microbial cultivability, has been rapidly applied in the field of aquatic microbiology as a useful tool for study LNA bacteria (Wang et al., 2009; Koch et al., 2014). Bacteria in the aquatic environment are generally divided into at least two groups by FCM analysis (Marie et al., 1997). The terms “Group I cells” and “Group II cells” were originally used to label the two groups (Li et al., 1995). Researchers later observed a correlation between fluorescence intensity and DNA/RNA content in the cells, and therefore renamed the two groups as “low-DNA (LDNA) bacteria” and “high-DNA (HDNA) bacteria,” respectively (Gasol et al., 1999). Lebaron et al. (2001) modified the designations of two groups again to “high nucleic acid-content (HNA) bacteria” and “low nucleic acid-content (LNA) bacteria” (Figure 1). Until now, the concept of LNA bacteria has remained confined to the field of FCM and it was a relative concept. Furthermore, scenarios have been proposed in which bacterial cells may switch between LNA and HNA bacteria (Bouvier et al., 2007).

### Cell Size

The characteristics of LNA bacteria were linked with the parameters in FCM. These links, including low fluorescence linked to low DNA content, low scatter linked to small cell size, filterability linked the small cell size, small cell sizes linked to small genome sizes and low DNA content, are supported by literature (Veldhuis et al., 1997; Gasol et al., 1999; Lebaron et al., 2002; Wang et al., 2009). When we link the concepts of LNA bacteria that can pass through 0.45-μm filter membrane to small-sized bacteria, such as: ultramicrobacteria, ultramicrocells, ultra-small bacteria, and nano-sized bacteria (Table 1), all these terms are within the scope of the definition of LNA bacteria. Accordingly, LNA bacteria is the most appropriate term to define such bacteria. Although all these names are vaguely defined based on isolated aspects of bacteria or different investigation techniques, they have the same properties, such as filterable (Proctor et al., 2018; Nakai, 2020). Additionally, from the perspective of cell volume, the cell volume of both LNA bacteria and small-sized bacteria is <0.1 μm^3^ (Table 2).

**Table 2:** An overview of LNA bacteria and their candidates in the environments.

Strains	Genus	Environment	Cell size	Genome size	References
*Aurantimicrobium photophilum* MWH-Mo1T	*Aurantimicrobium*	freshwater	0.052 μm^3^	1.75 Mb	Hahn et al., 2003, 2021
*Aurantimicrobium* sp. MWH-Mo2	*Aurantimicrobium*	freshwater	0.057 μm^3^	n.d.	Hahn et al., 2003
*Aurantimicrobium* sp. MWH-Mo3	*Aurantimicrobium*	freshwater	0.056 μm^3^	n.d.	Hahn et al., 2003
*Aurantimicrobium* sp. MWH-Po1	*Aurantimicrobium*	freshwater	0.058 μm^3^	n.d.	Hahn et al., 2003
*Aurantimicrobium* sp. MWH-Wo1	*Aurantimicrobium*	freshwater	0.054 μm^3^	n.d.	Hahn et al., 2003
*Aurantimicrobium* sp. MWH-Bo1	*Aurantimicrobium*	freshwater	0.061 μm^3^	n.d.	Hahn et al., 2003
*Aurantimicrobium* sp. MWH-Ta1	*Aurantimicrobium*	freshwater	0.051 μm^3^	n.d.	Hahn et al., 2003
*Aurantimicrobium* sp. MWH-Ta2	*Aurantimicrobium*	freshwater	0.056 μm^3^	n.d.	Hahn et al., 2003
*Aurantimicrobium* sp. MWH-Ta3	*Aurantimicrobium*	freshwater	0.083 μm^3^	n.d.	Hahn et al., 2003
*Aurantimicrobium minutum* KNCT	*Aurantimicrobium*	freshwater	0.04–0.05 μm^3^	1.62 Mb	Nakai et al., 2015, 2016
*Aquiluna borgnonia* 15G-AUS-rotT	*Aquiluna*	freshwater	Length: 0.62 μm Width: 0.45 μm	1.4 Mb	Pitt et al., 2021
*Chryseobacterium greenlandense* UMB10	*Chryseobacterium*	Greenland ice core	<0.1 μm^3^	n.d.	Miteva and Brenchley, 2005; Loveland-Curtze et al., 2010
*Chryseobacterium solincola* NF4	*Chryseobacterium*	sediments	0.004–0.02 μm^3^	~1.7 Mb	Suzina et al., 2011
*Chryseobacterium solincola* NF5	*Chryseobacterium*	sediments	0.004–0.04 μm^3^	~1.7 Mb	Suzina et al., 2011
*Kaistia* sp. NF1	*Kaistia*	soil	<0.1 μm^3^	~2.4 Mb	Duda et al., 2009
*Oxalicibacterium solurbis* MY14T	*Oxalicibacterium*	soil	~0.07 μm^3^	n.d.	Sahin et al., 2010
*Polynucleobacter* sp. CB	*Polynucleobacter*	freshwater	<0.05 μm^3^	2.05 Mb	Wang et al., 2009
*Polynucleobacter* sp. GS	*Polynucleobacter*	freshwater	<0.05 μm^3^	n.d.	Wang et al., 2009
*Polynucleobacter* sp. WW	*Polynucleobacter*	freshwater	<0.05 μm^3^	n.d.	Wang et al., 2009
*Polynucleobacter cosmopolitanus* MWH-MoIso2T	*Polynucleobacter*	freshwater	Length: 0.5–1.2 μm Width: 0.3–0.5 μm	1.78 Mb	Hahn et al., 2010
*Polynucleobacter victoriensis* MWH-VicM1T	*Polynucleobacter*	freshwater	Length: 0.4–1.1 μm Width: 0.3–0.5 μm	1.63 Mb	Hahn et al., 2010 Hahn et al., 2017
*Polynucleobacter hirudinilacicola* MWH-EgelM1-30-B4T	*Polynucleobacter*	freshwater	Length: 0.5–1.2 μm Width: 0.3–0.5 μm	2.01 Mb	Hahn et al., 2018
*Polynucleobacter campilacus* MWH-Feld-100 T	*Polynucleobacter*	freshwater	Length: 0.5–2.4 μm Width: 0.3–0.7 μm	1.98 Mb	Hahn et al., 2018
*Polynucleobacter paneuropaeus* MG-25-Pas1-D2T	*Polynucleobacter*	freshwater	Length: 0.6–1.2 μm Width: 0.3–0.6 μm	1.83 Mb	Hoetzinger et al., 2019
*Polynucleobacter paneuropaeus* MWH-UK1W16	*Polynucleobacter*	freshwater	Length: 0.4–1.2 μm Width: 0.3–0.6 μm	1.79 Mb	Hoetzinger et al., 2019
*Polynucleobacter paneuropaeus* MWH-Creno-4B4	*Polynucleobacter*	freshwater	Length: 0.4–0.9 μm Width: 0.3–0.5 μm	1.61 Mb	Hoetzinger et al., 2019
*Sphingopyxis alaskensis* RB2256	*Sphingopyxis*	marine	0.05–0.09 μm^3^	3.35 Mb	Schut et al., 1997
*Rhodoluna lacicola* MWH-Ta8T	*Rhodoluna*	freshwater	0.05 μm3	1.43 Mb	Hahn et al., 2014
*Rhodoluna limnophila* 27D-LEPIT	*Rhodoluna*	freshwater	Length: 0.49 μm Width: 0.28 μm	1.40 Mb	Pitt et al., 2019
*Rhodoluna limnophila* 1B-Mac	*Rhodoluna*	freshwater	Length: 0.44 μm Width:0.29 μm	1.42 Mb	Pitt et al., 2019
*Rhodoluna limnophila* 36A-HELLB	*Rhodoluna*	freshwater	Length: 0.46 μm Width: 0.27 μm	1.36 Mb	Pitt et al., 2019
*Candidatus* Fonsibacter ubiquis LSUCC0530	*Candidatus* Fonsibacter	marine	0.1 μm^3^	1.16 Mb	Henson et al., 2018
*Candidatus* Pelagibacter ubique HTCC1062	*Candidatus* Pelagibacter	marine	0.014 μm^3^	1.3 Mb	Ghuneim et al., 2018
*Candidatus* Planktophila rubra IMCC25003	*Candidatus* Planktophila	freshwater	0.041 μm^3^	1.35 Mb	Kim et al., 2019
*Candidatus* Planktophila aquatilis IMCC26103	*Candidatus* Planktophila	freshwater	0.061 μm^3^	1.46 Mb	Kim et al., 2019

n.d, no data.

### Physiological Properties

Wang et al. (2009) found that LNA bacteria can be effectively separated by 0.45-μm membrane filtration and widely exists in oligotrophic environments. Thus, LNA bacteria is also called oligotrophic bacteria and filterable bacteria. This will be described in detail in the following sections.

## The Characteristics of LNA bacteria

### Small Cell Size

The most striking property of LNA bacteria is their small cell size. Previous studies have found that the cell sizes of LNA bacterial communities in marine environment ranged from 0.035 to 0.06 μm^3^ (Huete-Stauffer et al., 2016). The biovolumes of LNA bacteria were lower than that of HNA bacteria in marine environment (García et al., 2014). For example, Morán et al. (2015) reported that the cell size of HNA bacteria (0.032–0.115 μm^3^; *n* = 114) was higher than that of LNA bacteria (0.050–0.056 μm^3^; *n* = 114). Proctor et al. (2018) found that LNA bacteria had an average diameter of 0.18 μm, an average length of 0.57 μm, and an average volume of 0.016 μm^3^ (*n* = 12). Additionally, the small cellular volume (0.009 ± 0.002 μm^3^) of representative strains such as Candidatus Katanobacteria (WWE3), Candidatus Microgenomates (OP11), and Candidatus Parcubacteria (OD1) were detected by cryogenic transmission electron microscopy (Luef et al., 2015). Ghai et al. (2013) reported that the cellular volume of ultra-small marine Actinobacteria was about 0.013 μm^3^ as estimated by flow cytometry-fluorescence *in situ* hybridization (FISH) techniques. The cellular volume of isolated LNA bacteria was generally found to be less than 0.1 μm^3^ (Table 2).

### Small Genomes

LNA bacteria not only possess small cellular volumes, but also small genomes (Table 2). Studies found that the DNA content of HNA bacteria was about 3.5 times higher than that of LNA bacteria (Schattenhofer et al., 2011), which can be reflected by the genome sizes of closely-related cultured representatives of HNA clades (3–6 Mb) and LNA clades (1.3–1.5 Mb). It was also reported that the typical representative LNA bacteria have small genome sizes, such as Candidatus Pelagibacter ubique HTCC1062 (1.54 Mb; Giovannoni et al., 2005), Candidatus Fonsibacter ubiquis LSUCC0530 (1.16 Mb; Henson et al., 2018), Candidatus Katanobacteria (WWE3; 0.88 Mb) and Candidatus Parcubacteria (OD1; 0.69 Mb; Kantor et al., 2013; Luef et al., 2015), while the representatives HNA bacteria such as Roseobacter or Flavobacteria have genome sizes ranging from 4.2 to 4.7 Mb (Pinhassi et al., 2006; Kalhoefer et al., 2011). Additionally, Candidatus Dependentiae (TM6), suggested as an LNA bacterial taxa previously had a small genomes (0.5–1.5 Mb; Longnecker et al., 2005; Proctor et al., 2018). And the genomes of SAR86 was modestly sized at 1.25–1.7 Mb (Dupont et al., 2012).

### Filterable

Microporous filtration (0.1–0.45 μm) is commonly used as a disinfection method for thermal agents and experimental solvents, and as a physical method for the removal of microorganisms such as bacteria in drinking water and wastewater treatment (Wang et al., 2007; Lee et al., 2010). The bacteria small enough to pass through a 0.45-μm membrane filter was termed filterable bacteria (Tabor et al., 1981). In the field of microbiology, it is also used to isolate and enrich LNA bacteria (Ma et al., 2019; Song et al., 2019a). Studies found that most of LNA bacteria are able to pass through a 0.45-μm filter while about 90% of the HNA bacteria were retained (Wang et al., 2009). Song et al. (2019a) found that the fraction of LNA bacteria in lake water samples significantly increased after 0.45-μm filtration. Thus, LNA bacteria was also called filterable bacteria. Studies found that the relative abundance of LNA bacteria was increased after filtration (Proctor et al., 2018), 15.53% ± 5.17% and 2.78% ± 0.88% increases for Actinobacteria and Candidatus Parcubacteria in LNA bacteria compared with the total bacteria (Song et al., 2019a).

It is worth noting that laboratory filtration experiments on a variety of different forms of bacteria have shown that the morphology of bacteria (including its flexibility) is the decisive factor in determining the passage of bacteria through the microporous membrane (Wang et al., 2008). Other factors that influence the passage of bacteria through the porous membrane include cell flexibility, microporous filtration rate, and microporous materials. Notably, the bacteria that can pass through the membrane are not necessarily LNA bacteria. There are two possible explanations for this. First, the cell volume and cell morphology of bacteria can change when they are confronted with conditions of low solutes undergo certain changes, which can allow these bacteria to pass through small pores (Tabor et al., 1981; Torrella and Morita, 1981). Second, some groups such as *Hylemonella gracilis*, due to the absence of a rigid cell wall, can effectively squeeze through small pores (Ghuneim et al., 2018).

### Oligotrophic

The main characteristics of oligotrophic environments are low energy flows and low absolute concentrations of nutrients (Egli, 2010). Nevertheless, such environments still contain 105–106 cells/ml bacteria (Morita, 1988). LNA bacteria are widespread in oligotrophic environments, including ocean, groundwater, tap water and alpine streams, and occupy a high proportion of the microbial communities (Wang et al., 2009). Studies reported that freshwater SAR11 bacteria (LD12) with the characteristics that slow but efficient uptake already at low substrate concentrations, enable them to adapt to oligotrophic habitats (Salcher et al., 2011). Consequently, LNA bacteria in such environments are considered to be “oligotrophic bacteria.” Two main physiological features of LNA bacteria enable them to survive in oligotrophic environments. One is their low growth rate. A previous study showed that the growth rate of Candidatus Pelagibacter ubique HTCC1062 was 0.40–0.58 cell divisions per day does not vary in response to nutrient addition (Giovannoni et al., 2005). Eguchi et al. (1996) also reported that the growth rate (0.13–0.16 h^−1^ at 23°C) of ultramicrobacteria (*Sphingopyxis alaskensis* RB2256) remains largely unchanged with carbon concentrations between 0.8 and 800 mg L^−1^, while the growth rate was 0.02–0.13 h^−1^ when cultured in glucose-limited media (Ostrowski et al., 2001). The second physiological feature is the adaptable surface-to-volume ratio. LNA bacteria can actively reduce their volume and increase the specific surface area to take up nutrients more efficiently in oligotrophic environments (Duda et al., 2012; Martínez-Cano et al., 2015; Liu et al., 2019).

## The Distribution and Diversity of LNA bacteria

### The Distribution of LNA Bacteria in the Natural Environment

LNA bacteria are widely distributed in various environments, including oceans, lakes, rivers, springs, groundwater, wetlands, and sediments (Servais et al., 2003; Longnecker et al., 2005, 2006; Suzina et al., 2011; Liu et al., 2017; Proctor et al., 2018; Song et al., 2019a; Eswaran and Khandeparker, 2021). The percentage of LNA bacteria in seawater and freshwater is summarized in Table 3. In marine environment, a relatively low proportion of LNA bacteria was reported in coastal seas, such as in NW Mediterranean coastal waters (42%–47%; Vila-Costa et al., 2012). In contrast, a higher proportion of LNA bacteria in open ocean was appeared than coastal or continental shelf communities (Servais et al., 2003; Longnecker et al., 2005, 2006), including the Southwest Atlantic Ocean (85%, Andrade et al., 2007), the North Atlantic Ocean (71%–85%, Schattenhofer et al., 2011). And in freshwater, the percentage of LNA bacteria ranges from 20% to 90% (generally >50%) of the total bacterial community, which varies with environmental conditions and ecosystem productivity (Bouvier et al., 2007; Wang et al., 2009; Proctor et al., 2018; Song et al., 2019a).

**Table 3:** Distribution of LNA bacteria in diverse ecosystems.

	Source	Percentage of small-sized bacteria	References
Seawater	North Atlantic	58%–64%	Li et al., 1995
	the eastern Mediterranean Sea	65%	Li et al., 1995
	Banyuls-sur-Mer	48.9%–51.4%	Lebaron et al., 2001
	the Leucate Lagoon	60.1%	Lebaron et al., 2001
	Coastal Canet Lagoon	57%	Servais et al., 2003
	the Banyuls-sur-Mer harbor	30%–36%	Servais et al., 2003
	the Gulf of Mexico	62%	Jochem et al., 2004
	the Arabian Sea	42%–54%	Zubkov et al., 2006
	the Southwest Atlantic Ocean	85%	Andrade et al., 2007
	the North Atlantic Ocean	71%–85%	Schattenhofer et al., 2011
	the Mediterranean Sea	40%–80%	Van Wambeke et al., 2011
	NW Mediterranean coastal	42%–47%	Vila-Costa et al., 2012
Freshwater	Tech River	26.5%–46.6%	Lebaron et al., 2001
	Tech river	38%	Servais et al., 2003
	Chriesbach stream	66%–70%	Wang et al., 2009
	Alpine stream	69%–79%	Wang et al., 2009
	Tap water	52%–54%	Wang et al., 2009
	Groundwater	72%–78%	Wang et al., 2009
	Lake Greifensee	18%–28%	Wang et al., 2009
	Wastewater effluent	56%–60%	Wang et al., 2009
	the Songhua River water	47%	Liu et al., 2017
	Groundwater	75%	Proctor et al., 2018
	River water	55%	Proctor et al., 2018
	Lake water	60%	Proctor et al., 2018
	Tap water	55%	Proctor et al., 2018
	Wastewater	84%	Proctor et al., 2018
	Lake water	40%–60%	Proctor et al., 2018
	River water	10%–60%	Sharuddin et al., 2018
	Lake water	40.9%–56.9%	Song et al., 2019a

### Phylogenetic Diversity of LNA Bacteria in the Natural Environment

LNA bacteria are the most abundant group of planktonic bacteria. Actinobacteria, Gammaproteobacteria were the dominant phyla, hgcI_clade and pseudomonas were the most abundant bacterial genera in LNA bacterial OTUs (Song et al., 2019a). The rare taxa, such as: Alphaproteobacteria—LD12, SAR11, SAR86, Candidatus Katanobacteria (WWE3), and Candidatus Microgenomates (OP11) were also found in LNA bacteria in freshwater environment (Proctor et al., 2018). In addition, representative bacteria such as Candidatus Parcubacteria (OD1; Nelson and Stegen, 2015), Candidatus Gracilibacteria (GN02: Rinke et al., 2013), Candidatus Saccharibacteria (TM7; He et al., 2015), Candidatus Dependentiae (TM6; McLean et al., 2013), and Candidatus Omnitrophica (OP3; Glöckner et al., 2010) were obtained through gene sequencing in LNA bacteria and cannot yet be cultured in the laboratory (Proctor et al., 2018). In marine environment, LNA bacteria was dominated by the Alphaproteobacterial clade SAR11 (45%–74%), Betaproteobacteria (2%–4%) in the North Atlantic Ocean (Schattenhofer et al., 2011). SAR11 and SAR86 contributed largely to LNA bacteria in NW Mediterranean coastal waters (Vila-Costa et al., 2012). And most of the isolated LNA bacteria belong to Alphaproteobacteria, Betaproteobacteria, and Actinobacteria (Table 2).

So far, the community composition of LNA and HNA bacteria remains controversial. Studies reported that LNA bacteria share the same clades with HNA bacteria in the Mediterranean coast (Servais et al., 2003). However, other studies have claimed that the phylogenetic composition varied between LNA and HNA bacteria. For example, the HNA bacteria was comprised of members of Bacteroidetes (26%–32%), Alphaproteobacteria without SAR11 (19%–25%), and Gammaproteobacteria without SAR86 (12% and 8%) at stations (BPLR and ARCT) in the North Atlantic Ocean (Schattenhofer et al., 2011), whereas the Alphaproteobacterial SAR11 clade (62%–72%) dominated in LNA bacteria (Mary et al., 2006; Schattenhofer et al., 2011). Additionally, a high abundance (>10%) of the Gammaproteobacterial clade SAR86 was reported in LNA bacteria of prokaryotic picoplankton in coastal seas (Zubkov and Sleigh, 1998; Zubkov et al., 2002). Members of Betaproteobacteria, Firmicutes, and Fibrobacter were also found in LNA bacteria (Vila-Costa et al., 2012). Interestingly, researchers found that some bacteria can shift between LNA and HNA bacterial groups in accordance with changes in external conditions. For example, Gammaproteobacteria, Actinobacteria, and Betaproteobacteria were equally likely to appear in both HNA and LNA bacteria in NW Mediterranean coastal waters (Vila-Costa et al., 2012). A similar phenomenon was also reported in freshwater environments where Methylobacteriaceae, Pseudomonas, and Alteromonodaceae were identified as both LNA (Longnecker et al., 2005) and HNA bacteria (Proctor et al., 2018). Additionally, previous studies found that the diversity indices of LNA bacteria were relatively lower than in HNA bacteria in both freshwater and marine environments (Vila-Costa et al., 2012; Song et al., 2019a). This may be due to HNA bacteria being composed of versatile fast growing bacteria (Schattenhofer et al., 2011), whose larger genomes allow them to occupy more ecological niches.

### The Impact of Environmental Factors on LNA Bacterial Community

LNA bacteria are sensitive to the living environment. Previous studies found that the distribution pattern of HNA and LNA bacteria in different marine and freshwater ecosystems was controlled by nutrients, substrate availability, pH, temperature, chlorophyll-a concentration and salinity (Joux et al., 2005; Nishimura et al., 2005; Bouvier et al., 2007; Belzile et al., 2008; Salcher et al., 2011; Liu et al., 2017; Song et al., 2019b). The net growth rates of LNA bacteria had a strong negative correlation with chlorophyll-a in temperate coastal waters (Huete-Stauffer and Morán, 2012). The decrease in cell size and nucleic acid content with temperature were also correlated, showing a common mean decrease of 0.4% per °C (Huete-Stauffer et al., 2016). The LNA/HNA ratios can shift with environmental conditions (Sharuddin et al., 2018), which lead to an exchange between LNA and HNA bacteria. The shifts in HNA and LNA bacterial fractions indicate the change in bacterial bulk activities as well as community composition. It was found that pH, total organic carbon (TOC), conductivity, and nitrogen-related concentrations played key roles in shaping LNA bacterial diversity (Song et al., 2019a). In addition, spatiotemporal changes also had significant effects on the cell granularity, DNA content, abundance, the distribution pattern and the community of LNA bacteria (Schattenhofer et al., 2011; Vila-Costa et al., 2012; Eswaran and Khandeparker, 2021).

## The Potential Metabolic Functions of LNA Bacteria

**Figure 2:**
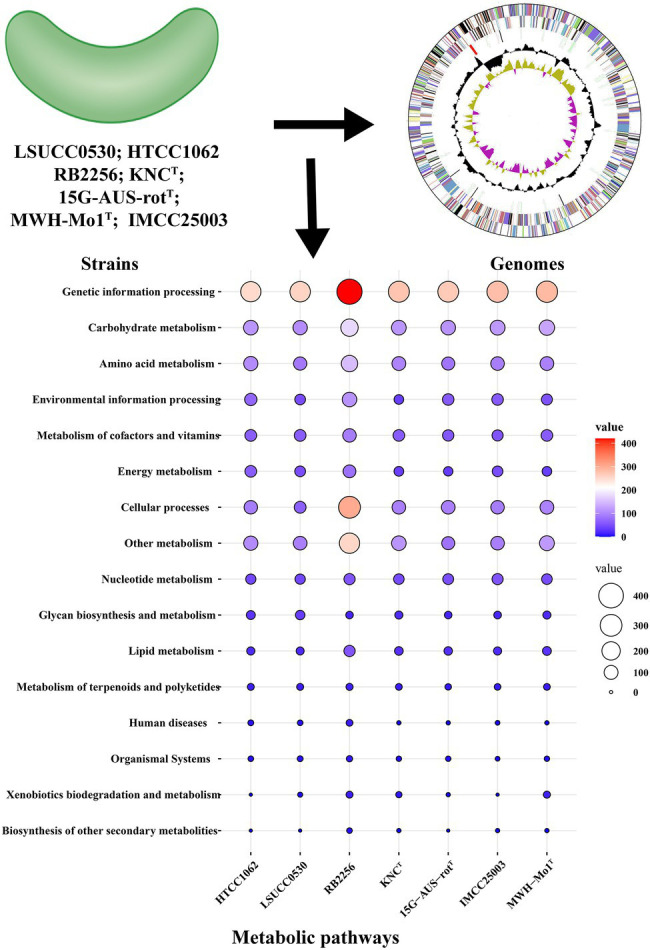
Analysis of the potential metabolic functions of LNA bacteria and ultramicrobacteria. The protein sequences of bacteria were obtained from NCBI database and annotated in KEGG database. The value in the diagram represents the number of proteins involved in the pathways. HTCC1062: Candidatus Pelagibacter ubique HTCC1062; LSUCC0530: Candidatus Fonsibacter ubiquis LSUCC0530; RB2256: *Sphingopyxis alaskensis* RB2256; KNCT: *Aurantimicrobium minutum* KNCT; 15G-AUS-rotT: *Aquiluna borgnonia* 15G-AUS-rotT; IMCC25003: Candidatus Planktophila rubra IMCC25003; MWH-Mo1T: *Aurantimicrobium photophilum* MWH-Mo1T.

**Figure 3:**
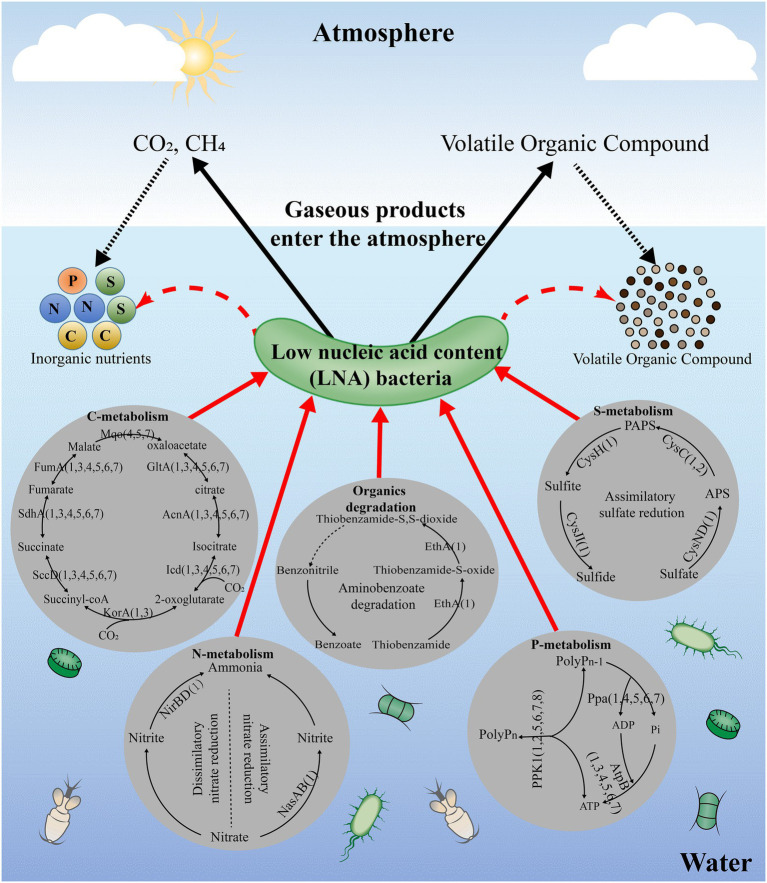
The potential metabolic functions of LNA bacteria and ultramicrobacteria in the aquatic environments. The enzymes in the figure means that can be annotated in pathways of the strains in KEGG and the number in the figure means the strains. All data used for the analysis were obtained from NCBI database; 1: *Sphingopyxis alaskensis* RB2256; 2: Candidatus Pelagibacter ubique HTCC1062; 3: Candidatus Fonsibacter ubiquis LSUCC0530; 4: *Aurantimicrobium minutum* KNCT; 5: *Aquiluna borgnonia* 15G-AUS-rotT; 6: Candidatus Planktophila rubra IMCC25003; 7: *Aurantimicrobium photophilum* MWH-Mo1T.

The predicted metabolic functions of isolated LNA bacteria were investigated using the KEGG database and numerous proteins were annotated in pathways linked to carbohydrate metabolism, energy metabolism and lipid metabolism, which means that LNA bacteria can participate in material circulation in the environment (Figure 2; Giovannoni, 2017; Henson et al., 2018; Song et al., 2019a). In particular, enzymes found in carbohydrate and amino acid metabolism enable LNA bacteria to use small molecular weight compounds such as carboxyl groups and amino acids as a carbon/energy source for survival in oligotrophic environments (Tripp et al., 2008; Sun et al., 2011). Comparative genomic analysis from different LNA bacteria showed that some metabolic pathways missed in the strains (Figure 3). This phenomenon has been reported in previous studies. For example, LD12 lacks the glyoxylate shunt and some single carbon (C1) metabolism. However, the EMP pathway was contained in the genomes of LD12 and was not found in most marine SAR11 (Eiler et al., 2016). Additionally, a complete assimilatory sulfate reduction pathway is missing in Candidatus Fonsibacter ubiquis LSUCC0530 (Henson et al., 2018). In addition, LNA bacteria play an important role in the degradation of organic matter. They had the potential to degrade aromatic compounds in freshwater (Song et al., 2019a) and oxidize many labile, low-molecular-weight organic compounds, including some volatile organic compounds (VOCs) and methylated compounds (Giovannoni, 2017). Ma et al. (2016) found that the typical LNA bacteria, Curvibacter sp. PAE-UM, is capable of degrading phthalate esters under oligotrophic conditions. More functions discussed in this review were predicted by omics techniques, although the concerted effect of the entire LNA bacterial community in ecosystem functions remains unknown. To fully understand the functional characteristics of small-sized bacteria, more small-sized bacteria need to be isolated and cultured.

## The Cultivation Strategy of LNA Bacteria

Although the unknown metabolic features of uncultured bacteria could be elucidated by metagenomics and single-cell genomics, the data are still difficult to interpret without fundamental knowledge of the underlying bacterial physiology. Without cultivation, many questions about the roles of uncultured bacteria in their natural settings remain unanswered (Lewis et al., 2020). To improve our understanding of the functions of uncultured bacteria, it is essential to increase our capacity for bringing the uncultured bacteria from the environment into culture.

**Figure 4:**
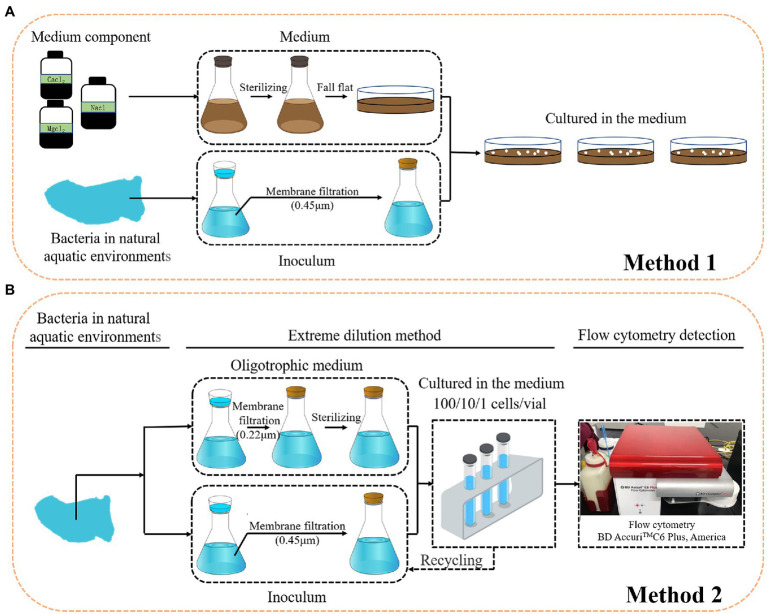
Cultivation strategies for LNA bacteria. **(A):** conventional cultivation; **(B):** dilution cultures, filtration and FCM.

Conventional cultivation methods are laborious, time consuming, and most importantly, selective and biased for the growth of specific microorganisms (Figure 4A; Zengler et al., 2002). Most cells obtained from nature and visualized by microscopy are viable, but the majority of the bacteria do not form visible colonies on plates. To cultivate the LNA bacteria from aquatic environments, many cultivation methods have been tested. First, dilution cultures. Dilution cultures were proposed by Button et al. (1993), who demonstrated that they have advantages for studying oligo-bacteria. This method relies on diluting samples in low nutrient media until the final dilution tube contains the most abundant members of the population which can grow in the media (Duda, 2011). The type strain of ultramicrobacteria named *Sphingopyxis alaskensis* RB2256 was isolated from Resurrection Bay in Alaska using dilution cultures (Schut et al., 1993). Second, filtration. Filtration was found to be a useful method for the isolation of LNA bacteria from the environments. Different ultramicrobacteria members also have been obtained through the filtration of environmental samples (Nakai, 2020). Additionally, to isolate more uncultured bacteria, researchers have tried to use cultivation media with a composition more suited to their natural environment (Connon and Giovannoni, 2002; Kaeberlein et al., 2002), such as filter-sterilized seawater supplemented with different concentrations of nutrients (Zengler et al., 2002). The advantage of this approach is that the conditions for bacterial growth are as natural as possible, even taking into account any alterations during experimental handling of seawater. Third, FCM. FCM is one approach that has been applied to the selection of non-cultivable microbes. The cell count in the marine and freshwater are barely detectable by conventional techniques. In such cases, FCM with the advantages, such as: being culture-independent, fast, sensitive, and accurate, are the most suitable quantitative techniques to study of non-cultivable microbes in aquatic environments (Wang et al., 2010). Hence, the combination of filtration for dilution cultures, cultivation media, and FCM facilitates the study of non-cultivable microbes in aquatic environments (Figure 4B). Such an approach was successfully applied in the isolation of LNA bacteria from freshwater (Wang et al., 2009), and in the cultivation of opportunistic pathogenic bacteria in oligotrophic aquatic environments (Vital et al., 2007).

## Future Perspectives

### Finding Suitable Cultivation Conditions

A comprehensive understanding of the microbial world undoubtedly requires cultivation, yet the difficulties associated with the isolation and cultivation of oligotrophic bacteria are well documented (Schut et al., 1997). A fundamental problem that limits their cultivation is that obligately oligotrophic bacteria are innately sensitive to nutrients (Cavicchioli et al., 2003). In addition, certain physiological groups of bacteria require specific growth conditions, including resuscitation from dormancy; symbiotic interdependencies; physical contact or spatial proximity; and physicochemical environmental conditions (Lewis et al., 2020). While the key physiological traits for some microorganisms can be revealed through the metatranscriptome and metaproteome, accurately determining microbial features such as growth kinetics, metabolic mechanisms, and physiological properties is incredibly difficult, as these cannot be inferred from genome sequences alone. Thus, it is essential to increase our capacity for bringing microorganisms from the environment into pure culture.

### Enrich LNA Bacterial Resources and Elucidate Its Metabolic Mechanism

LNA bacteria are an important part of marine microbial community, with the potential to be metabolically active and contributing toward mineralization in the oligotrophic marine environment (Cavicchioli et al., 2003). To some extent, bacteria with a genome size as low as <1 Mb have certain physiological and metabolic defects. For example, several carbon degradation pathways in SAR11 were lost in freshwater habitats (Eiler et al., 2016). Very limited information is available on the mechanism of adaptation by LNA bacteria, especially in oligotrophic environments. Cultivation of representative LNA bacteria is important to reveal their physiological and metabolic properties.

### Explore the Ecological Function of LNA Bacteria

Groups that are highly abundant in an environment are likely to have pivotal ecological roles (Lewis et al., 2020). Many uncultivated microorganisms preserve a lot of new genes with unknown functions under extreme environmental conditions. Although the function of many microorganisms can be predicted by means of omics, these omics approaches cannot replace culture-based phylogenetic and functional studies, since functional prediction relies heavily on the availability of well-annotated genomes from cultures. Therefore, more in-depth investigations are needed to explore the functional properties of small-sized bacteria in the ecosystem.

## Conclusion

In this work, the characteristics, biodiversity, and cultivation strategy of LNA bacteria were reviewed. The main conclusions are:

- Various terminologies have been used to define LNA bacteria, and all of these terms are within the scope of the definition of LNA bacteria.
- LNA bacteria not only have a small cell size and small genome, but also have filterable and oligotrophic characteristics.
- LNA bacteria are the main group of planktonic bacteria and are widely distributed in various environments. The distribution pattern and the composition of LNA bacteria can shift with environmental factors.
- Prediction of the metabolic functions of LNA bacteria revealed that LNA bacteria can participate in material cycling in the environment. However, to fully understand the functional characteristics of LNA bacteria, more LNA bacteria need to be isolated and cultured.
- For isolating pure LNA bacteria, a combination of filtration for dilution cultures, cultivation media, and FCM is considered to be effective.

## Author Contributions

WH: data curation and writing—original draft preparation. HZ: visualization. XL: data curation. RL: software. MB: writing—review and editing. YW: supervision, writing—review and editing, and funding acquisition. All authors contributed to the article and approved the submitted version.

## Funding

This work was supported by the National Key Research and Development Program of China (2020YFC1807000), the National Natural Science Foundation (31870485), the Tianjin Natural Science Foundation (19JCZDJC39600), and 111 program, Ministry of Education, China (T2017002).

## Conflict of Interest

The authors declare that the research was conducted in the absence of any commercial or financial relationships that could be construed as a potential conflict of interest.

## Publisher’s Note

All claims expressed in this article are solely those of the authors and do not necessarily represent those of their affiliated organizations, or those of the publisher, the editors and the reviewers. Any product that may be evaluated in this article, or claim that may be made by its manufacturer, is not guaranteed or endorsed by the publisher.
